# Treatment of Intestinal Inflammation With Epicutaneous Immunotherapy Requires TGF-β and IL-10 but Not Foxp3^+^ Tregs

**DOI:** 10.3389/fimmu.2021.637630

**Published:** 2021-02-26

**Authors:** Xin Chen, M. Cecilia Berin, Virginia L. Gillespie, Hugh A. Sampson, David Dunkin

**Affiliations:** ^1^Division of Pediatric Gastroenterology and the Mindich Child Health and Development Institute, The Icahn School of Medicine at Mount Sinai, New York City, NY, United States; ^2^Division of Pediatric Allergy and Immunology, Precision Immunology Institute and Mindich Child Health and Development Institute, The Icahn School of Medicine at Mount Sinai, New York City, NY, United States; ^3^Center for Comparative Medicine and Surgery, The Icahn School of Medicine at Mount Sinai, New York City, NY, United States; ^4^DBV Technologies, LLC, Montrouge, France

**Keywords:** epicutaneous immunotherapy, regulatory T cells, tolerance, colitis, IBD, immunotherapy

## Abstract

**Background:** Inflammatory bowel disease (IBD) involves an increase in T effector cells in the intestines that disrupts the normal balance with T regulatory cells (Tregs). A therapy that restores this balance has the potential to treat IBD. We have shown that epicutaneous exposure to OVA induces Tregs that are able to induce tolerance. The Tregs also migrate to the intestines where they alleviate colitis in mice, demonstrating the potential for skin induced Tregs to treat intestinal inflammation. We investigated the role of Foxp3, IL-10, and TGF-β in the suppression of colitis by epicutaneous immunotherapy (ET).

**Methods:** RAG1^−/−^ mice were transferred with CD4^+^CD45RB^hi^ T cells from wild type mice to induce colitis. To determine whether Foxp3^+^ Tregs, IL-10-, or TGF-β-producing Tregs were necessary, Foxp3-DTR, IL-10^−/−^, or CD4-dnTGFBRII mice were immunized with OVA and OVA TCR enriched T cells were added. As control groups, some mice were given OVA TCR enriched T cells from wild type mice or no OVA TCR enriched T cells. Half of the mice in each group were then exposed on the skin to Viaskin patches containing OVA weekly for 3 weeks. Mice given OVA TCR enriched T cells from Foxp3-DTR mice were given diphtheria toxin (DT) or not in addition to ET. Mice were assessed for weight loss, colon length, colonic cytokine production, and histological inflammation.

**Results:** ET, after injection with OVA TCR enriched T cells derived from wild type mice, prevented weight loss, decreased colonic inflammatory cytokine production and histological colitis. ET in the absence of the OVA TCR enriched T cells did not alleviate colitis. ET, after injection with OVA TCR enriched T cells derived from Foxp3-DTR mice, prevented weight loss, decreased colonic inflammatory cytokine production, and histological colitis. Ablation with DT did not impair the ability of ET to alleviate colitis. ET failed to alleviate colitis when OVA TCR enriched T cells were derived from IL-10^−/−^ or CD4-dnTGFBRII mice.

**Conclusions:** ET through induction of Tregs, which produce IL-10 and TGF-β, could be a promising treatment for IBD.

## Introduction

Inflammatory bowel disease (IBD), which consists of Crohn's disease (CD) and ulcerative colitis (UC), is a chronic inflammatory gastrointestinal disease. With the rapid growth of industrial society, the incidence of IBD has increased widely across developed countries (1). It affects over 2 million individuals in North America, and 3.2 million in Europe. Newly industrialized countries also have an increasing prevalence of IBD, making it a major public health problem in the world (2). Abdominal pain, growth failure, nausea, diarrhea, vomiting, anorexia, rectal bleeding, and weight loss are common symptoms of IBD. The current medical treatment of IBD includes salicylates, corticosteroids, immune-suppressants (thiopurine analogs and methotrexate), biologics, and surgery based upon the severity and extent of the disease. Many of these therapies work by suppressing aspects of the immune system and can thus have potentially severe side effects, such as myelosuppression, pancreatitis, hepatitis, and an increased risk of malignancies or infections (3). Thus, there is an urgent need to develop new therapies to mitigate or eliminate these potentially serious side effects.

It is believed that IBD is caused by abnormal immune responses to environmental factors and/or intestinal microbiota in genetically predisposed individuals (4). Under normal conditions, people develop immune tolerance in which T regulatory cells (Tregs) play a critical role to help prevent autoimmunity, induce tolerance against dietary antigens, protect against commensal bacteria in the intestine, and suppress allergy and pathogen-induced immunopathology (5). However, in IBD patients, there is an increase in T effector cells that disrupts the normal balance resulting in many more effector T cells as compared to Tregs (6, 7). A therapy that could restore this balance may have the potential to treat IBD.

Many studies have sought to remedy autoimmune diseases, like rheumatoid arthritis and encephalomyelitis, by inducing oral tolerance which is actively mediated by Tregs (8, 9). However, IBD patients have defective oral tolerance responses (10). It is, therefore, necessary to find alternative routes of tolerance induction to treat IBD. Several studies have shown the skin to be a highly active immune organ through which tolerance induction by epicutaneous application can be used to treat food allergies and various autoimmune diseases such as experimental autoimmune encephalomyelitis and collagen-induced arthritis (11–13). In our previous study, we demonstrated that epicutaneous immunotherapy (ET) induced the formation of Tregs that could migrate to the small intestine and colon and could block subsequent immune reactions (14). This tolerance was dependent on transforming growth factor beta (TGF-β) (14). We then showed that intestinal inflammation in different mouse models can be abrogated by ET using a Viaskin® patch containing the model antigen ovalbumin (OVA) (14). In the DSS colitis and SAMP/YITFc ileitis models, ET alleviated colitis without any additional manipulation. In contrast, the CD45RB^hi^ model required the addition of OVA specific T cells because RAG1^−/−^ mice do not produce mature T or B cells and the injected population of CD45RB^hi^ (naïve T cells) cells is small (3.5 × 10^5^) and thus unlikely to contain any T cells that would recognize OVA. In the CD45RB^hi^ T cell transfer model, there was increased expression of Foxp3, TGF-β1 and TGF-β2 along with increases in CD4^+^CD25^+^Foxp3^+^ and CD4^+^CD25^+^LAP^+^ T cells in the colons of treated mice. However, the mechanism of how ET alleviated colitis was not clear.

Tregs are necessary to maintain tolerance with commensal bacteria and innocuous food antigens found in the gut. The transcription factor forkhead box protein P3 (Foxp3) is an important marker for Tregs, as many studies have shown that defective Foxp3 leads to lethal immune dysregulation (15, 16). Importantly, gut-homing Foxp3 Tregs are required for intestinal tolerance in the lamina propria. IL-10 is an important cytokine produced by a large number of Tregs in the gut and helps to inhibit immune responses and maintain immune tolerance (17). Its importance in the intestinal immune milieu is apparent in both mice and humans as IL-10 deficient mice develop spontaneous inflammation of the colon, and humans with mutations in IL-10 or IL-10 receptor (IL-10R) also develop colitis at an early age (18). Another important immunosuppressive cytokine secreted by Tregs is TGF-β which can suppress Th1 and Th2 cells. TGF-β deficient mice develop spontaneous colitis and dysregulated TGF-β signaling is observed in IBD patients (19). Both IL-10 and TGF-β have been shown to be necessary cytokines to help maintain peripheral and intestinal tolerance (20, 21).

Here we utilized the adoptive T cell transfer colitis model that uses RAG1^−/−^ mice, which do not produce mature T and B cells, and determined which immunosuppressive elements involved in achieving tolerance were necessary for ET to suppress colonic inflammation. This model was chosen due to the ease of altering the various populations of cells from which ET induces Tregs that can alleviate colitis. As an extension of our previous studies and given the relevance of the CD4^+^CD45RB^hi^ T cell transfer model of colitis to human disease, we examined the necessity of Foxp3, IL-10, and TGF-β in suppressing colitis in mice and show that ET required the presence of TGF-β and IL-10 but not Foxp3^+^ Tregs to alleviate colitis.

## Materials and Methods

### Mice

RAG1^−/−^ (CD45.2), C57BL/6 (CD45.1 and CD45.2), Foxp3-GFP-DTR, IL10^−/−^, and CD4-dnTGFBRII mice were obtained from the existing colonies at The Icahn School of Medicine at Mount Sinai which were originally purchased from Jackson Laboratories (Bar Harbor, ME). Experimental mice were age and gender matched in each experimental group and a mix of both males and females was used. All experiments were repeated at least twice to confirm any results. All mice were housed with food and water *ad libitum*, 12 h light/dark cycle, and 20 ± 2°C room temperature. All procedures and protocols were approved by the Icahn School of Medicine at Mount Sinai Institutional Animal Care and Use Committee (IACUC).

### CD4^+^CD45RB^hi^ T Cell Transfer Colitis Model

Naïve CD4^+^ T cell were isolated from C57BL/6 (CD45.1) mouse spleens and lymph nodes with a mouse CD4^+^ T cell isolation kit as per the manufacturer's instructions (Stemcell Technology, Canada). The isolated CD4^+^ T population was labeled with CD45Rb-FITC, CD62L-PE, and CD4-APC (eBiosciences, San Diego, CA). CD4^+^CD62L^+^CD45RB^hi^ T cells were sorted by flow cytometry. Then 3.5 × 10^5^ cells were injected into RAG1^−/−^ mice by intraperitoneal (IP) injection. Mouse body condition, the form of their stool and weight were monitored at least weekly. Mice started to lose weight and develop looser stools around 3–4 weeks in our facility indicating that colitis was present.

### OVA TCR Enriched T Cell Transfer

To ensure the presence of OVA-specific T cells in the model, our original model utilized an additional transfer of CD4^+^ T cells from OT-II/RAG^−/−^ mice a day prior to beginning exposure with OVA-Viaskin (14). Here wild-type [C57BL/6 (CD45.2)], Foxp3-GFP-DTR, IL10^−/−^, or CD4-dnTGFBRII mice were immunized with OVA and OVA TCR enriched T cells from these mice were used in place of the OTII/RAG^−/−^ cells so that we could utilize the various knockouts in the model. The immunization was performed by IP injection of 200 μL of 1 mg/mL OVA mixed with Alum in a 1:1 ratio weekly for 2 weeks. This was followed by gavage feeding of 1 mg OVA with 10 μg cholera toxin weekly for 2 weeks. Immunization served to induce OVA TCR enriched T cells that could be a source of OVA responsive T cells which could be induced by ET to form OVA-specific Tregs. T cells from immunized mouse spleens and lymph nodes were isolated using Stemcell Technology mouse memory CD4^+^ T cells isolation kit (Stemcell Technology). The isolated CD4^+^ CD62L^−^CD44^hi/int^ T cell subpopulation was then injected into the mice that had shown symptoms consistent with the development of colitis (week 3–4). As a control experiment, mice were not given OVA TCR enriched T cells but still treated as if they had been given OVA TCR enriched T cells.

### Ablation of Foxp3^+^ Tregs

Foxp3-GFP-DTR mice on a C57BL/6 background (22) were used as noted above (23). In the colitic mice that received OVA TCR enriched T cells from these mice, experimental groups were injected with 75 ng per gram body weight of diphtheria toxin (DT) (Sigma) to deplete any OVA-specific Foxp3^+^ Tregs induced by each application of Viaskin. Control mice were not given DT. Depletion of Foxp3^+^ cells was confirmed by flow cytometry 48 h after injection using antibodies against CD4 and CD25 and examining CD4^+^CD25^+^ cells for GFP positivity by flow cytometry.

### Epicutaneous Exposure

The day after the OVA TCR enriched T cells were injected into the RAG1^−/−^ mice with colitis, all the mice were anesthetized and dorsal fur removed with depilatory cream (Veet; Reckitt Benckiser, Parsippany, NJ), which has been shown to not induce inflammation in prior studies (14). Mice were then exposed to Viaskin® (DBV Technologies, Bagneux, France), which has a central transparent plastic membrane with an electrically charged polyethylene coating. The antigen protein is maintained on the membrane by electrostatic forces and then solubilized and released when attached to skin (24). The treatment group received Viaskin containing 100 μg OVA. The control group received Viaskin containing vehicle alone. Both groups were treated with corresponding patches weekly for 48 h for 3 consecutive weeks (14). After the last Viaskin application, all the mice were gavage fed with 1 mg OVA to increase gut homing and activation of Tregs. Two weeks later all mice were sacrificed.

### Histological Scoring

Colons were cut longitudinally, flattened, rolled and then fixed in 4% formalin. Two complete cross sections of each Swiss roll were stained with hematoxylin-eosin (H&E). One cross-section was then assessed for the severity of colitis by a pathologist blinded to the treatment group. The extent of involvement of the epithelium, mucosa, submucosa and muscularis were scored. A total score was calculated by adding the individual scores for a maximum of 20 (25).

### Cytokine Measurement

A piece of colon tissue (0.5 × 0.5 cm) was taken from the same portion of colon of each mouse and cultured overnight in RPMI with 1 × protease inhibitors (Thermo Fisher Scientific, MA) and 1 × phosphatase inhibitors (Thermo Fisher Scientific, MA). Cytokine secretion was measured in the supernatant (IL-10, IL-17A, TNF-α, IFN-γ, IL-6, IL-4, IL-2) by Cytometric Bead Array (CBA) using a Th1/Th2/Th17 kit (BD Biosciences, San Jose, CA) per the manufacturer's instructions. Samples were acquired using a Cytoflex flow cytometer (Beckman Coulter life sciences, Indianapolis, IN) and data was analyzed with FlowJo software (Becton, Dickinson & Company, Franklin Lakes, NJ).

### Quantitative Real-Time (RT) PCR

RNA was extracted from the same part each colon sample using TRIZOL (Invitrogen, Carlsbad, CA) followed by isopropanol precipitation. RNA was then reverse transcribed to complementary DNA (cDNA) using a PrimeScript^TM^ RT Reagent Kit (TaKaRa, Mountain View, CA). Real-time PCR was performed using SYBR^TM^ Green Master Mix (Thermo Fisher Scientific, Fair lawn, NJ) as previously described. The sequences of primers were listed in Supplementary Table 1. The target gene mRNA expression was normalized to the control group and calculated using the ^ΔΔ^CT method.

### Statistics

The differences between 2 groups were analyzed by Mann–Whitney *t*-test, and comparisons between multiple groups were done using one-way ANOVA. This was followed by either non-parametric Mann–Whitney *U*-test or Bonferroni analysis when appropriate. All data were analyzed by using Prism software (GraphPad, San Diego, CA). The results are presented as the mean ± SD with a *P* < 0.05 considered as statistically significant different. *P*-values are indicated by ^*^P < 0.05, ^**^P < 0.01, ^***^P < 0.001.

## Results

### ET Alleviated Colitis When OVA TCR Enriched T Cells From Wild-Type Mice Were Present but Not in Their Absence

**Figure 1:**
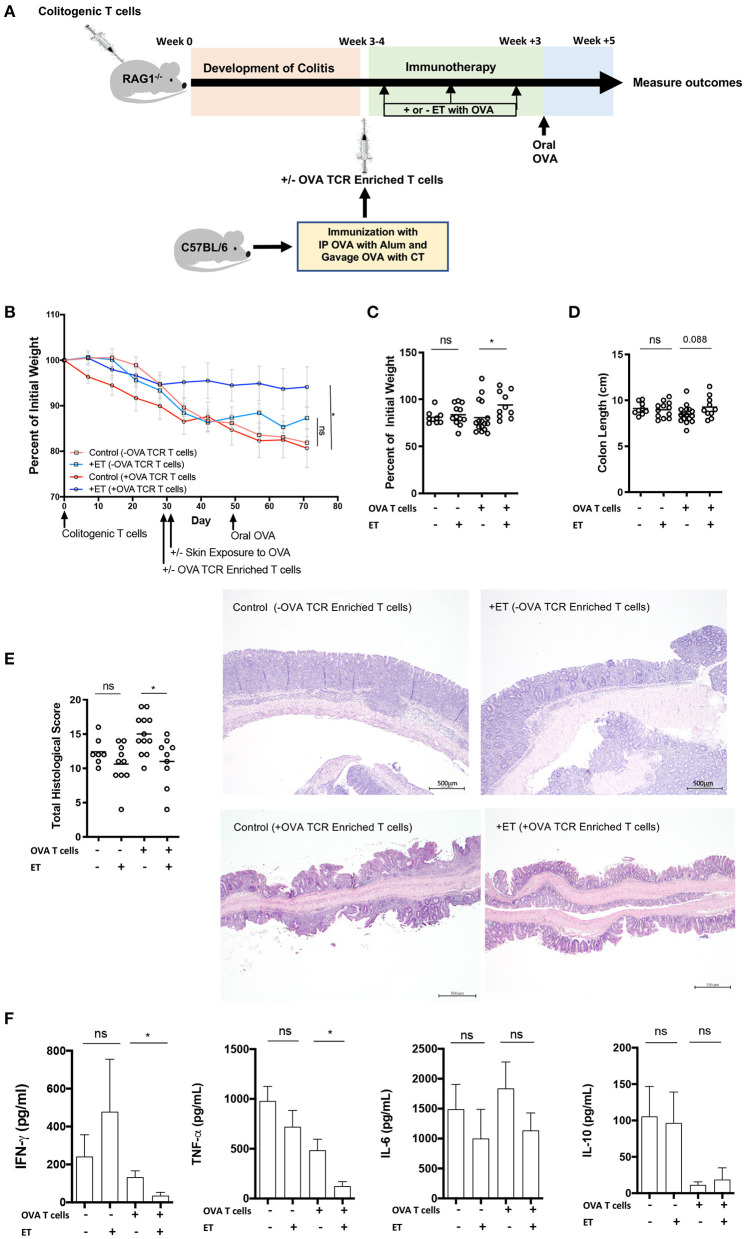
ET abrogated colitis when OVA TCR enriched T cells from wild-type mice were present but not in their absence. **(A)** Schematic demonstrating the design of the experiments: ET, Epicutaneous immunotherapy. RAG1^−/−^ mice were injected with colitogenic T cells (CD4^+^CD45RB^hi^) from wild-type mice. Once mice exhibited symptoms (weight loss, loose stool, or blood in the stool) of colitis at week 3 or 4, mice were injected or not injected with OVA TCR enriched T cells from C57BL/6 mice that were immunized with IP OVA with alum followed by gavage feeding with OVA with cholera toxin (CT). After this injection, mice were exposed on the skin (+ET) with Viaskin containing OVA or vehicle alone (–ET) weekly for 3 weeks. All mice then received an oral dose of OVA given by gavage. **(B)** The percentage of initial body weight of mice with colitis with (+OVA T cells) or without (–OVA T cells) the addition of C57BL/6 OVA TCR enriched T cells and exposed to OVA-Viaskin (+ET) or not (control). **(C)** The final percentage of initial body weight as measured when sacrificing them. **(D)** Colon length of the mice after sacrificing them. **(E)** Histological score of colonic tissue as determined by a pathologist blinded to the treatment group. Representative H&E sections of colon at 40× magnification demonstrating the control group that did not receive OVA TCR enriched T cells [control (–OVA TCR Enriched T cells)] and the treated group that did not receive OVA TCR enriched T cells [+ET(–OVA TCR Enriched T cells)] with similar inflammation (total histological scores of 12 and 11, respectively), with diffuse infiltration of the colonic mucosa and expansion of the submucosa by numerous inflammatory cells with necrosis and loss of mucosal epithelium (erosions) and loss of crypts. The inflammatory cells are a mixture of lymphocytes, plasma cells, and lesser numbers of neutrophils. The control sample that did received OVA TCR enriched T cells [Control (+OVA TCR Enriched T cells)] had diffuse infiltration of the colonic mucosa and submucosa by numerous inflammatory cells with loss of mucosal epithelium (erosions), loss of crypts, distortion of the remaining crypts, and a few crypt abscesses. The inflammatory cells are a mixture of lymphocytes, plasma cells, and lesser numbers of neutrophils. The treated sample that received OVA TCR enriched T cells [+ET (+OVA TCR Enriched T cells)] shows multifocal infiltration of the colonic mucosa and submucosa by mild numbers of inflammatory cells composed mainly of lymphocytes and plasma cells. The total histological score of the representative section of control and +ET that received OVA TCR enriched T cells were 14 and 10, respectively. **(F)** Cytokine production by cultured colon samples (2 pooled experiments of 5–8 mice/group; *p < 0.05; ns, not significant).

In our previous study, the CD4^+^CD45RB^hi^ transfer model was utilized with the addition of T cells from OT-II/RAG^−/−^ mice to ensure the presence of OVA-specific T cells in the model. ET treated mice had less severe colitis as evidenced by reduced weight loss, colonic inflammation, and production of inflammatory cytokines (TNF-α, IFN-γ, and IL-17A) from the colon (14). Here, the CD4^+^CD45RB^hi^ transfer model was used but with the addition of OVA TCR Enriched T cells (CD4^+^CD62L^−^CD44^hi/int^) from OVA immunized wild type mice [C57BL/6 (CD45.2)] instead of T cells from OT-II/RAG^−/−^ mice. This was done to set the stage for using various knockout models as the source of OVA specific T cells without having to generate new strains of mice. Upon development of colitis mice were injected or not with OVA TCR enriched T cells. Then treated mice were exposed to Viaskin containing OVA while control mice were exposed to Viaskin with vehicle alone. All mice then received a one-time oral dose of OVA to ensure gut homing of T cells and their activation (Figure 1A).

In the mice given OVA TCR enriched T cells, ET significantly reduced weight loss (Figures 1B,C). Colon shortening which occurs with colonic inflammation trended toward being significantly reduced in the ET group (Figure 1D). Total histological scores were reduced in the ET group compared to controls (Figure 1E). The decrease in histologic score in the ET group is apparent in the representative H&E slides showing the control sample with diffuse infiltration of the colonic mucosa and submucosa by numerous inflammatory cells with loss of mucosal epithelium (erosions), loss of crypts, distortion of the remaining crypts, and a few crypt abscesses. The ET group shows less inflammation with multifocal infiltration of the colonic mucosa and submucosa by mild numbers of inflammatory cells (Figure 1E). The production of the inflammatory cytokines TNF-α and IFN-γ, were also decreased in the ET group (Figure 1F). IL-6 and IL-10 production were not statistically different between the groups (Figure 1F). IL-2, IL-4, and IL-17A were not detectable (data not shown). Regulatory factors were examined by rtPCR and not found to be significantly different in their expression between the ET group and controls (Supplementary Figure 1A). Like our original studies, these data demonstrate that ET can alleviate colitis.

To demonstrate that ET is affecting cells within the injected OVA TCR enriched T cell population and thereby alleviating colitis, mice were not given OVA TCR enriched T cells and ET was performed or not. In the absence of OVA TCR enriched T cells, there were no differences between ET and control groups in all parameters including body weight (Figures 1B,C) and colon length (Figure 1D). Histological scores were similar as demonstrated by representative H&E slides showing the control group and ET group with similar diffuse infiltrations of the colonic mucosa and expansion of the submucosa by numerous inflammatory cells with necrosis and loss of mucosal epithelium (erosions) and loss of crypts (Figure 1E). There were no differences in colon cytokine production (Figure 1F) and regulatory element expression (Supplementary Figure 1B). This indicates that OVA TCR enriched T cells are necessary for ET to alleviate colitis in our model and that this group of cells is the source of the Tregs that alleviate colitis.

### ET Alleviated Colitis Even in the Absence of Foxp3^+^ Tregs

**Figure 2:**
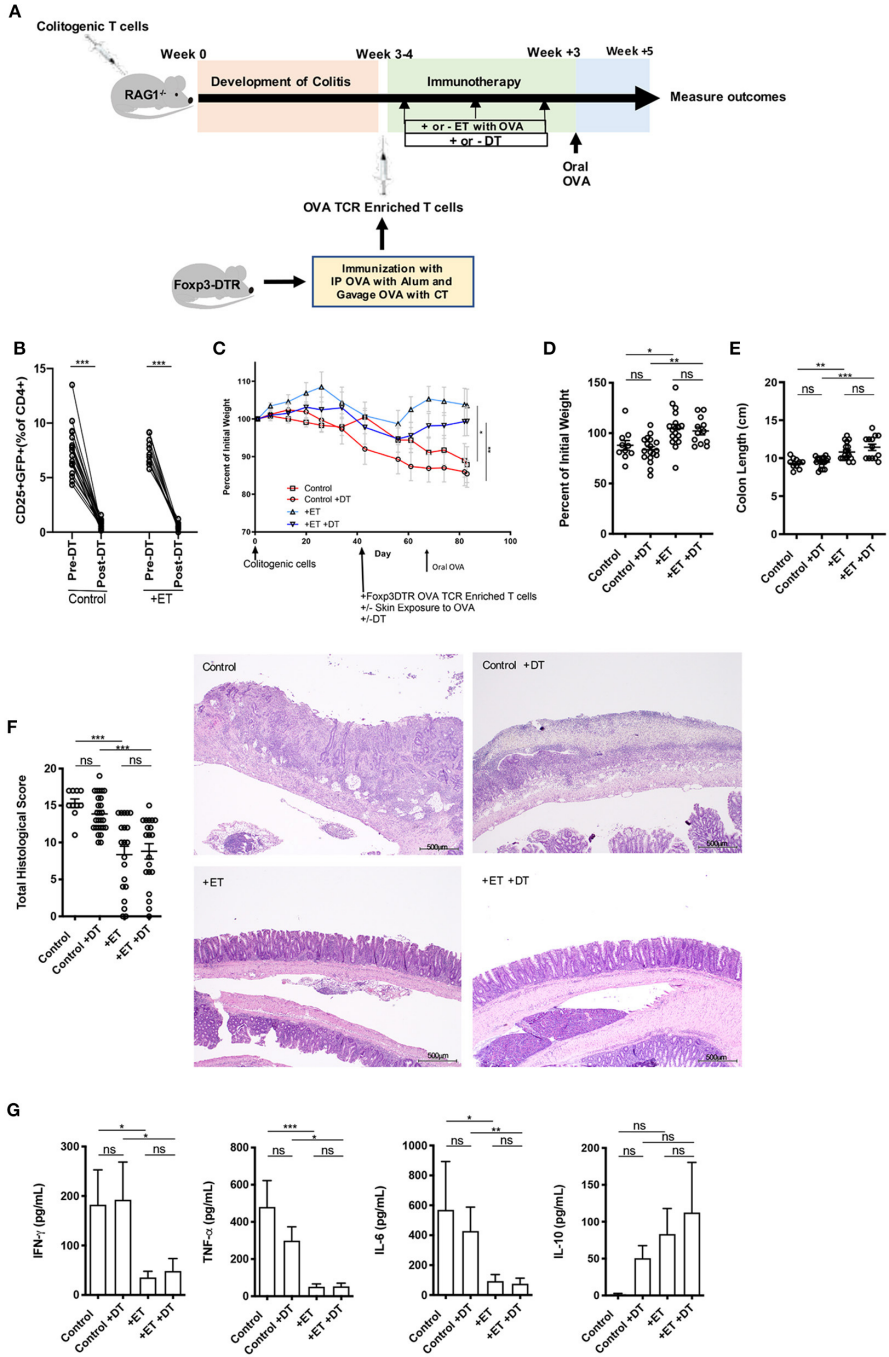
Induced Foxp3^+^ Tregs were not necessary for ET to abrogate colitis. **(A)** Schematic demonstrating the design of the experiments: ET, Epicutaneous immunotherapy. RAG1^−/−^ mice were injected with colitogenic T cells (CD4^+^CD45RB^hi^) from wild-type mice. Once mice exhibited symptoms (weight loss, loose stool, or blood in the stool) of colitis at week 3 or 4, mice were injected with OVA TCR enriched T cells from Foxp3-DTR mice that were immunized with IP OVA with alum followed by gavage feeding with OVA with cholera toxin (CT). After this injection, mice were exposed on the skin (+ET) with Viaskin containing OVA or vehicle alone (–ET) weekly for 3 weeks. Mice were then injected or not with DT to deplete any induced Foxp3^+^ T cells. All mice then received an oral dose of OVA given by gavage. **(B)** Depletion of Foxp3^+^ T cells after administration of DT. **(C)** The percentage of initial body weight of mice with colitis induced via the CD4^+^CD45RB^hi^ transfer with the addition of OVA TCR enriched T cells from Foxp3-DTR mice without or with DT (+DT) and exposed to OVA-Viaskin (+ET) or not (control). **(D)** The final percentage of initial body weight as measured when sacrificing them. **(E)** Colon length of the mice after sacrificing them. **(F)** Histological score of colonic tissue as determined by a pathologist blinded to the treatment group. Representative H&E sections of colon at 40× magnification that demonstrate both control groups (control and control+DT) with areas of complete loss of mucosa (ulceration) with replacement by abundant inflammatory cells that also infiltrate the submucosa and muscularis and both treated groups (+ET and +ET+DT) with less inflammation and preservation of normal architecture including an intact mucsoca. The total histological score of the representative section of control, control+DT, +ET, and +ET+DT were 17, 17, 10, and 11, respectively. **(G)** Cytokine production by cultured colon samples (3 pooled experiments of 4–5 mice/group; *p < 0.05, **p < 0.01, ***p < 0.001, ns, not significant).

Here, we used the CD4^+^CD45RB^hi^ T cell transfer model where RAG1^−/−^ mice were injected with OVA TCR enriched T cells (CD4^+^CD62L^−^CD44^hi/int^) from Foxp3-GFP-DTR mice instead of from C57BL/6 mice and were treated or not with DT to deplete any induced Foxp3^+^ T cells after each exposure to Viaskin with OVA (Figure 2A). Depletion was confirmed by flow cytometry on peripheral blood (Figure 2B and Supplementary Figure 2). All mice then received a one-time oral dose of OVA to ensure gut homing of T cells and their activation (Figure 2A).

Control groups lost weight to an equal extent regardless of whether Foxp3^+^ Tregs were ablated or not. ET significantly reduced body weight loss in the presence or absence of Foxp3^+^ Tregs (Figures 2C,D). Colon shortening which occurs with colonic inflammation was significantly reduced in ET groups independent of Foxp3^+^ Treg depletion (Figure 2E). Consequently, total histological scores were reduced in the ET groups compared to control groups (Figure 2F). The histology is apparent in the representative H&E slides with both control groups with areas of complete loss of mucosa (ulceration) with replacement by abundant inflammatory cells that also infiltrate the submucosa and muscularis and both ET groups with less inflammation and preservation of normal architecture including an intact mucosa (Figure 2F). The production of the inflammatory cytokines TNF-α, IFN-γ, and IL-6 from colons were decreased in the ET groups independent of Foxp3^+^ Treg depletion (Figure 2G). IL-10 production was not statistically different between the groups (Figure 2G) and IL-2, IL-4, and IL-17A were not detectable (data not shown). Expression of TGF-β1 and TGF-β2 were increased in ET groups as compared to controls but Foxp3 and IL-10 were not significantly different between the two groups (Supplementary Figure 1C). These data indicate that even in the absence of induced Foxp3^+^ Tregs, ET could alleviate colitis.

### IL-10 Was Necessary for ET to Alleviate Colitis

**Figure 3:**
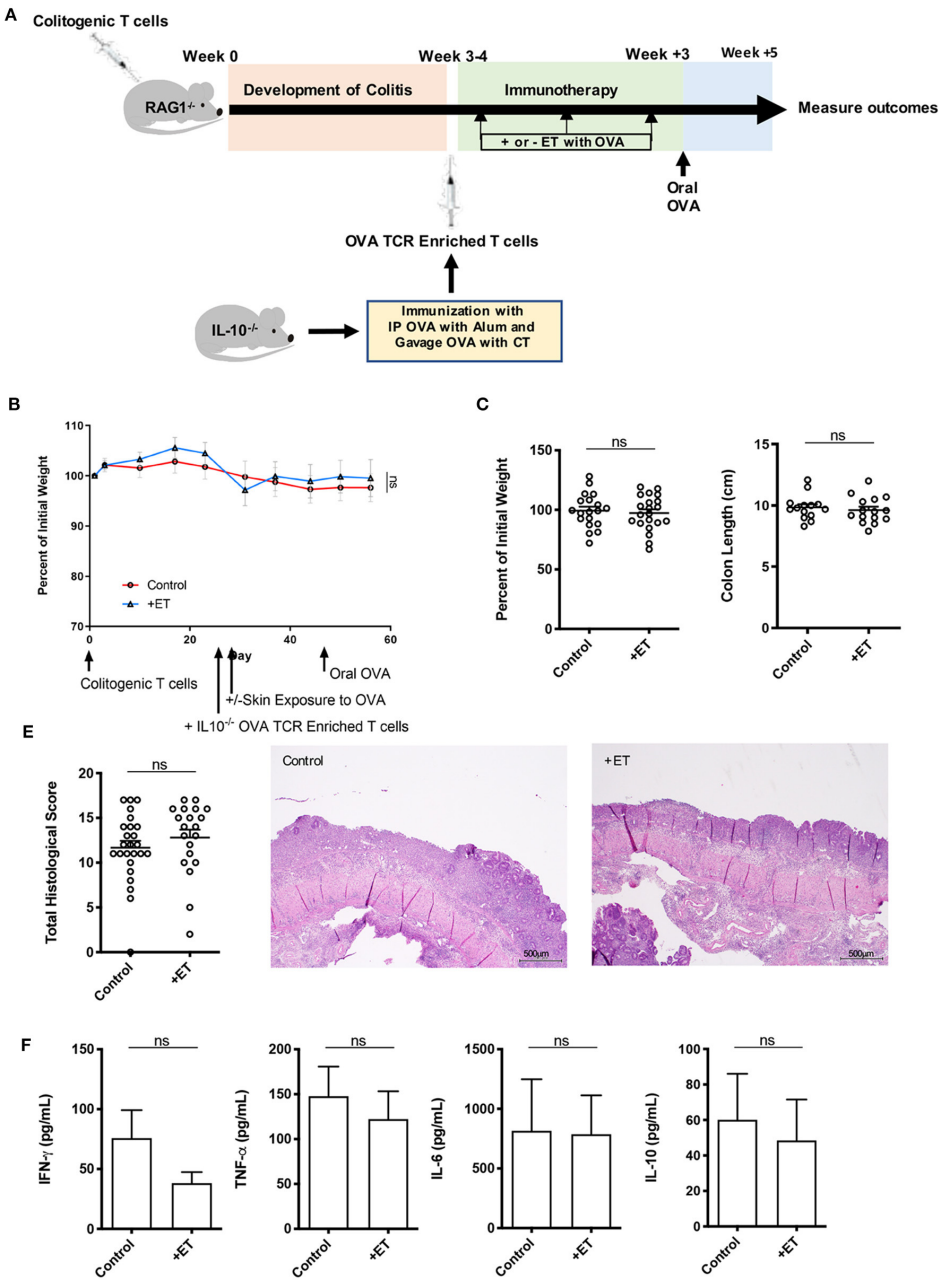
IL-10 was necessary for ET to abrogate colitis. **(A)** Schematic demonstrating the design of the experiments: ET, Epicutaneous immunotherapy. RAG1^−/−^ mice were injected with colitogenic T cells (CD4^+^CD45RB^hi^) from wild-type mice. Once mice exhibited symptoms (weight loss, loose stool, or blood in the stool) of colitis at week 3 or 4, mice were injected with OVA TCR enriched T cells from IL-10^−/−^ mice that were immunized with IP OVA with alum followed by gavage feeding with OVA with cholera toxin (CT). After this injection, mice were exposed on the skin (+ET) with Viaskin containing OVA or vehicle alone (–ET) weekly for 3 weeks. All mice then received an oral dose of OVA given by gavage. **(B)** The percentage of initial body weight of mice with colitis induced via the CD4^+^CD45RB^hi^ transfer with the addition of OVA TCR enriched T cells from IL10^−/−^ mice and then exposed to OVA-Viaskin (+ET) or not (control). **(C)** The final percentage of initial body weight as measured when sacrificing them. **(D)** Colon length of the mice after sacrificing them. **(E)** Histological score of colon samples as determined by a pathologist blinded to the treatment group. Representative H&E sections of colon at 40x magnification that demonstrate both control and treated groups (control and +ET) with areas of complete loss of mucosa (ulceration) with replacement by abundant inflammatory cells that also infiltrate the submucosa and muscularis. The total histological score of the representative section of control and +ET were 15 and 15, respectively. **(F)** Cytokine production by cultured colon samples (3 pooled experiments of 4–5 mice/group; ns, not significant).

To determine the role of IL-10 in ET mediated protection from colitis, RAG1^−/−^ mice in the CD4^+^CD45RB^hi^ T cell transfer model were injected with OVA TCR enriched T cells (CD4^+^CD62L^−^CD44^hi/int^) from IL-10^−/−^ mice prior to initiating ET. After the injection of OVA TCR enriched T cells, treated mice were exposed to Viaskin containing OVA while control mice were exposed to Viaskin with vehicle alone. All mice then received a one-time oral dose of OVA to ensure gut homing of T cells and their activation (Figure 3A).

In these experiments, mice had minimal weight loss but still exhibited significant histological inflammation indicating that they had colitis. There were no differences between ET and control groups in all parameters including body weight (Figures 3B,C) and colon length (Figure 3D). Histological scores were similar and are apparent in the representative H&E slides showing both control and ET groups with areas of complete loss of mucosa (ulceration) with replacement by abundant inflammatory cells that also infiltrate the submucosa and muscularis (Figure 3E). There were no differences in colon cytokine production (Figure 3F) and colon regulatory expression (Supplementary Figure 1D). This indicates that IL-10 was necessary for ET to alleviate colitis in this model.

### TGF-β Was Necessary for ET to Alleviate Colitis

**Figure 4:**
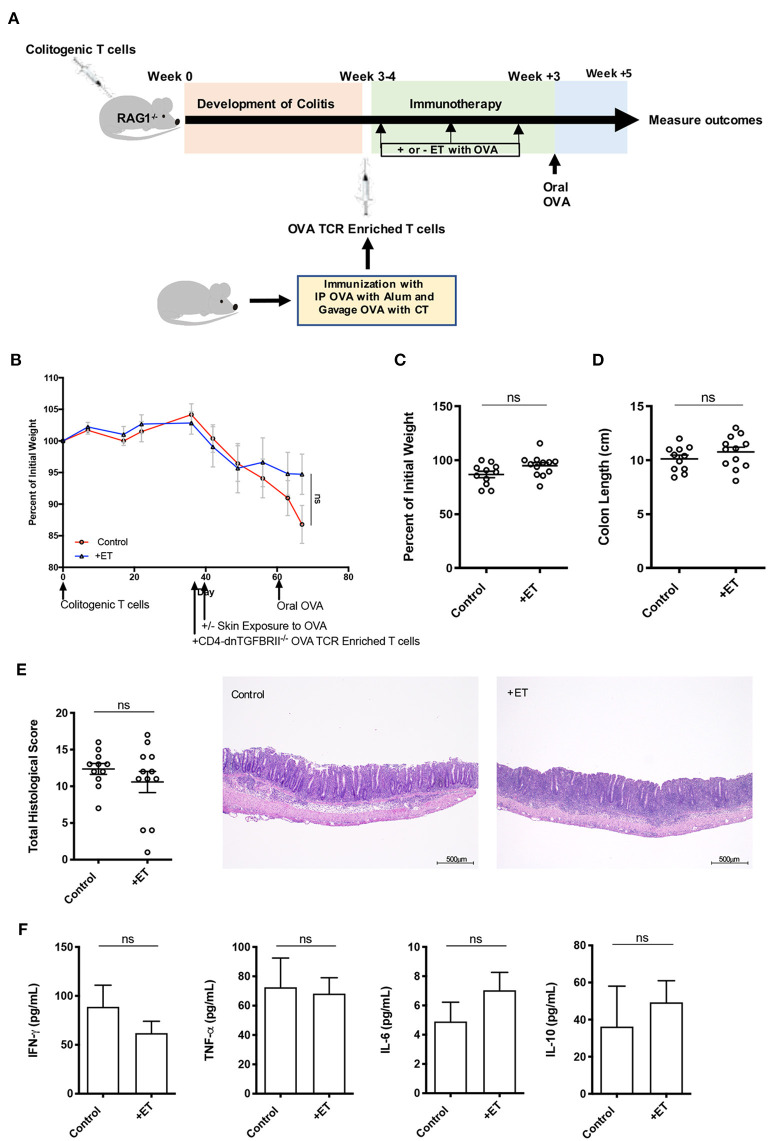
TGF-β was necessary for ET to abrogate colitis. **(A)** Schematic demonstrating the design of the experiments: ET, Epicutaneous immunotherapy. RAG1^−/−^ mice were injected with colitogenic T cells (CD4^+^CD45RB^hi^) from wild-type mice. Once mice exhibited symptoms (weight loss, loose stool, or blood in the stool) of colitis at week 3 or 4, mice were injected with OVA TCR enriched T cells from CD4-dnTGFBRII mice that were immunized with IP OVA with alum followed by gavage feeding with OVA with cholera toxin (CT). After this injection, mice were exposed on the skin (+ET) with Viaskin containing OVA or vehicle alone (-ET) weekly for 3 weeks. All mice then received an oral dose of OVA given by gavage. **(B)** The percentage of initial body weight of mice with colitis induced via the CD4^+^CD45RB^hi^ transfer with the addition of OVA TCR enriched T cells from CD4-dnTGFBRII mice and exposed to OVA-Viaskin (+ET) or not (control). **(C)** The final percentage of initial body weight as measured when sacrificing them. **(D)** Colon length of the mice after sacrificing them. **(E)** Histological score of colon samples as determined by a pathologist blinded to the treatment group. Representative H&E sections of colon at 40× magnification that demonstrate both control and treated groups (control and +ET) with erosions, loss of crypts, an expansion of the submucosa and infiltration of the muscularis by inflammatory cells. The total histological score of the representative section of control and +ET were 13 and 14, respectively. **(F)** Cytokine production by cultured colon samples (3 pooled experiments of 4–5 mice/group; ns, not significant).

To understand the implication of TGF-β in this process, the same CD4^+^CD45RB^hi^ T cell transfer model was employed where RAG1^−/−^ mice were injected with OVA TCR enriched T cells (CD4^+^CD62L^−^CD44^hi/int^) from CD4-dnTGFBRII mice that have a T cell-targeted inactivation of TGF-β prior to ET. After the injection of OVA TCR enriched T cells, treated mice were exposed to Viaskin containing OVA while control mice were exposed to Viaskin with vehicle alone. All mice then received a one-time oral dose of OVA to ensure gut homing of T cells and their activation (Figure 4A).

There were no differences between ET and control groups in all parameters including body weight (Figures 4B,C) and colon length (Figure 4D). Histological scores were similar in control and ET groups as demonstrated by the representative H&E slides showing both groups with erosions, loss of crypts, an expansion of the submucosa and infiltration of the muscularis by inflammatory cells (Figure 4E). There were no differences in colon cytokine production (Figure 4F) and colon regulatory expression (Supplementary Figure 1E). This indicates that TGF-β was necessary for ET to alleviate colitis in this model.

## Discussion

In this study, we verified first that ET alleviates colitis in the adoptive transfer model. Since our goal was to examine the mechanism by which this occurs by looking at the necessity of Foxp3^+^ Tregs and the regulatory cytokines IL-10 and TGF-β in our model, we first had to determine if the added memory T cells from OVA immunized mice were the source of the Tregs that were alleviating colitis. We found that without the addition of memory T cells (OVA TCR enriched T cells), colitis was not alleviated in the treated mice. This indicates that Tregs which are alleviating colitis are being formed within this population and not from the initial transfer of naïve T cells. We then found that both regulatory cytokines, IL-10 and TGF-β, played an essential role in this process, but induced Foxp3^+^ Tregs were dispensable.

Using Tregs for immunological therapy has been studied in many fields, such as solid organ transplantation, type 1 diabetes mellitus, and systemic lupus erythematosus (26). For IBD, studies in murine models have shown that the adoptive transfer of Tregs will suppress intestinal inflammation and alleviate symptoms (27). Similar studies in humans have also shown that giving Tregs to CD patients can alleviate symptoms (28). CD patients have a defect in oral tolerance induction (10). Therefore, it is necessary to look for other routes to achieve immunotherapy, which have high response rates and are easy to access. As a highly active immunologic organ, the skin may induce protection, sensitization, and tolerance. A previous study for food allergy treatment using ET with Viaskin found that antigen was taken up by Langerhans cells and dermal dendritic cells, which when depleted block the induction of Tregs (29). Epicutaneous Viaskin application in a murine allergy model generated gut-homing LAP^+^Foxp3^−^ Tregs that could suppress food-induced anaphylaxis (30). Our previous study demonstrated that ET treatment with Viaskin-OVA prevented or halted intestinal inflammation and there was an increase in expression of Foxp3 and TGF-β and an increase in CD4^+^CD25^+^Foxp3^+^ and CD4^+^CD25^+^LAP^+^ T cells in the intestines of treated mice (14). Our current study demonstrates that while an increase in Foxp3 may help alleviate colitis, it is not necessary. On the other hand, the increases in TGF-β and LAP^+^ T cells in the intestines of treated mice appear to be indispensable to ET's ability to treat colitis.

Our initial investigations examining the ability of ET to block subsequent immune responses contrasted with the mechanisms understood in oral tolerance that show a requirement for Foxp3^+^ Tregs (14, 31). Like our previous findings, we found that ET could alleviate colitis independent of the presence of Foxp3^+^ Tregs. These results were surprising given that peripherally induced Foxp3^+^ Tregs have been shown to be necessary for the induction of oral tolerance (31). Additionally, mutations in Foxp3 can lead to severe food allergies and the development of two severe autoimmune syndromes: XLAAD (X-linked autoimmunity-allergic dysregulation syndrome) and IPEX (immunodysregulation, polyendocrinopathy, enteropathy, X-linked syndrome) in which oral tolerance is abrogated (32, 33). Without Foxp3, ET could still suppress colonic inflammation. This indicated that tolerance through the skin does not require Foxp3 and that it was mediated by other Tregs. This makes sense given that expression of TGF-β1 and TGF-β2 in the colons of the treated mice was increased. Other researchers have also found that Foxp3 was not essential for immuno-suppressive activity. For example, in one experiment monospecific CD4^+^ T cells were transferred to RAG1^−/−^ mice and recolonizing cells did not express significant Foxp3, but had acquired a regulatory capacity (34).

Given the ability to alleviate colitis independent of the presence of Foxp3^+^ Tregs, Tregs that can secrete TGF-β and mediate oral tolerance were likely involved (35). Our initial investigations examining the ability of ET to block subsequent immune responses were similar to oral tolerance in regards to the requirement of TGF-β (14) and this again held true for the ability of ET to alleviate colitis. TGF-β has multiply functions in regulation of T cell proliferation and differentiation. The CD4-dnTGFbRII mouse strain used in this study has a T cell-targeted inactivation of TGF-β that leads to spontaneous colitis with a massive infiltration of lymphocytes and activation of T cells in the intestine and other organs (36). The absence of TGF-β in T cells is known to lead to reductions in CD4^+^Foxp3^+^ Tregs as evident in another conditional knockout, CD4-Cre Tgfbr2^fl/fl^ mice (19). This suggests that TGF-β signaling is necessary for maintaining the population of Tregs and for intestinal tolerance. Recent evidence points to the essential and non-redundant role of TGF- β in peripheral tolerance (37). In addition, TGF- β (and IL-10) can inhibit antigenic presentation to stimulate the generation of tolerogenic dendritic cells that can lead to the production of Tregs (38). In IBD, TGF-β appears to be important in the pathogenesis and therapies that target restoration of TGF-β could suppress inflammation. One example is a Smad7 antisense oligonucleotide which inhibits Smad7 and has been shown to restore TGF-β-induced Smad3 phosphorylation in CD and UC (39).

In our previous experiments, we found that the ability of ET to block systemic immune responses was not impaired when IL-10 was neutralized by IL-10R antibody (14). However, in this study, IL-10 was necessary for ET to alleviate colitis. This indicates that IL-10 was not necessary for ET to block systemic immune responses but is necessary in the gut for ET to alleviate colitis. This is concordant with what is known about the role of IL-10 in the gut and its role in the prevention of colitis. IL-10^−/−^ mice develop an inflammatory infiltrate consisting of lymphocytes, macrophages and neutrophils in the colon around 4–8 weeks of life due to failure to regulate Th1-mediated responses (40). In one murine study, a cell population, which produces IL-10 but does not express Foxp3 could inhibit T cell proliferation (41). In another experiment, T cells from transgenic mice expressing a dominant-negative IL-10 receptor specifically in T cells (CD4-dominant negative IL-10R transgenic mice) were transferred into a colitis mouse model, and similar to our experiments, they found that IL-10R^impaired^ Tregs failed to block colitis compared with the wild type Tregs (42). Humans with IL-10 or IL-10R mutations also suffer from severe colitis at early age (18). These all indicate the important role of IL-10 in intestinal immune responses.

Our study has several limitations. First, the study sought to determine the necessity of various regulatory elements in the ability of ET to treat colitis. In doing so, we utilized the CD45RB^hi^ transfer model and that necessitated a complicated protocol. The model required the addition of OVA TCR enriched T cells from which OVA-specific Tregs could develop, thus our need to immunize mice and inject memory T cells into the model prior to ET. A more straightforward model would eliminate any confounders that this might have caused. In our initial studies, we used multiple models, two (DSS and SAMP/YITFc) of which did not require the addition of OVA-specific T cells, but these models do not lend themselves well to studying the regulatory elements involved. Second, in the Foxp3 experiments we confirmed depletion of Foxp3^+^ T cells by examining the peripheral blood and this might not represent what is occurring in the intestines. Prior studies, however, have shown that depletion in the peripheral blood correlated with that in the spleen, lymph nodes and intestines (43). In this model, we also ablated Foxp3^+^ T cells that were induced by ET but conventional Foxp3^+^ T cells may have been present within the OVA TCR enriched T cell population. The conventional Foxp3^+^ T cells likely played little role in alleviating inflammation given that control groups also received OVA TCR enriched T cells and still had significant histological inflammation that was similar to that seen in our experiments done with CD4^+^ T cells from OTII/RAG mice (14). Finally, we inferred that the mechanism involves both IL-10 and TGF-β based upon the inability of ET to alleviated colitis when OVA TCR enriched T cells came from IL-10 and TGF-β knockout mice. This result ideally should be confirmed in experiments where each cell type is isolated and demonstrated to restore ET responsiveness.

In summary, the intestinal immune environment in IBD is unbalanced with excessive and continuous immune responses to antigenic triggers that lead to structural and functional damage of the intestine. Current treatments for IBD have various degrees of potential side effects due to their focus on suppressing immune responses. A therapy that can augment the suppressive or Treg responses, could minimize side effects and be much safer by inducing patients' own Tregs and thus reestablishing a balance in the intestinal immune system. Our study verifies this concept by inducing Tregs with ET in a colitis mouse model and differentiating some of the regulatory elements that may be involved in helping Tregs to alleviate colitis. We found that ET could alleviate colitis in the adoptive T cell transfer model of colitis if TGF-β and IL-10 were present but Foxp3^+^ Tregs were dispensable. Thus, ET has potential to augment Tregs to suppress inflammation in the intestine and could be a potential treatment strategy for IBD.

## Data Availability Statement

The raw data supporting the conclusions of this article will be made available by the authors, without undue reservation.

## Ethics Statement

The animal study was reviewed and approved by Icahn School of Medicine at Mount Sinai Institutional Animal Care and Use Committee.

## Author Contributions

XC, MB, and DD were involved in the design of the experiments. XC, VG, and DD performed the experiments and analyzed the data. XC, MB, VG, HS, and DD contributed extensively to the writing and editing of the manuscript. All authors approved the final manuscript.

## Conflict of Interest

HS is a consultant for DBV Technologies. The remaining authors declare that the research was conducted in the absence of any commercial or financial relationships that could be construed as a potential conflict of interest.
