# Supplementary material for: Treatment of Intestinal Inflammation With Epicutaneous Immunotherapy Requires TGF-β and IL-10 but Not Foxp3+ Tregs
Source: Front Immunol. 2021 Feb 26;12:637630. doi: 10.3389/fimmu.2021.637630 (PMC7952322; doi:10.3389/fimmu.2021.637630)
Supplement: Supplementary file 1 [file Data_Sheet_1.docx]

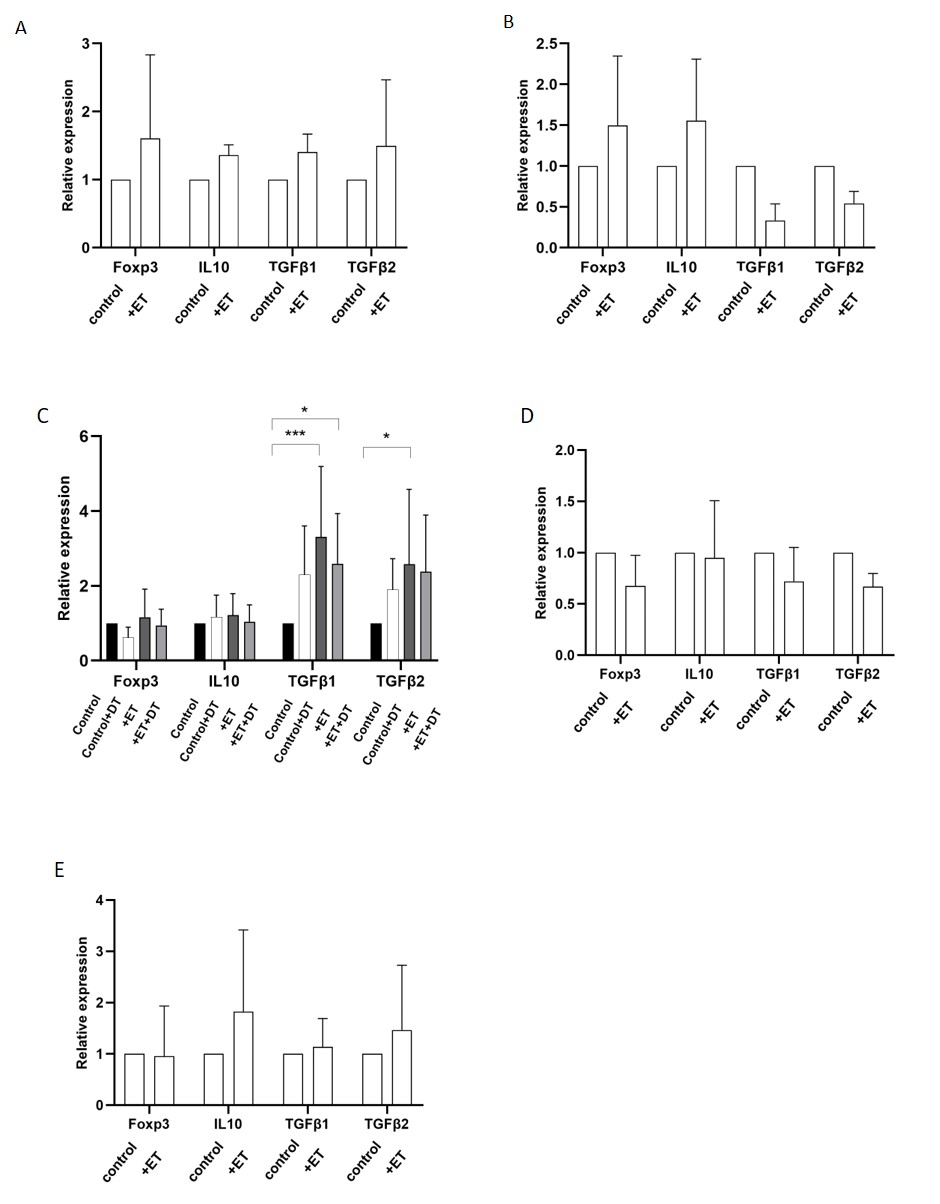


**Figure S1**. **Expression of regulatory genes of the colon as measured by RT-PCR.** (**A)** Expression of regulatory genes from experiments with wild type OVA TCR enriched T cells. (**B)** Expression of regulatory genes from experiments without the addition of OVA TCR enriched T cells. (**C)** Expression of regulatory genes from experiments with OVA TCR enriched T cells from Foxp3-DTR mice with or without the addition of DT. (**D)** Expression of regulatory genes from experiments with OVA TCR enriched T cells from IL-10 knockout mice. (**E)** Expression of regulatory genes from experiments with OVA TCR enriched T cells from TGF-β knockout mice. *p<0.05, **p<0.01, ***p<0.001.

**
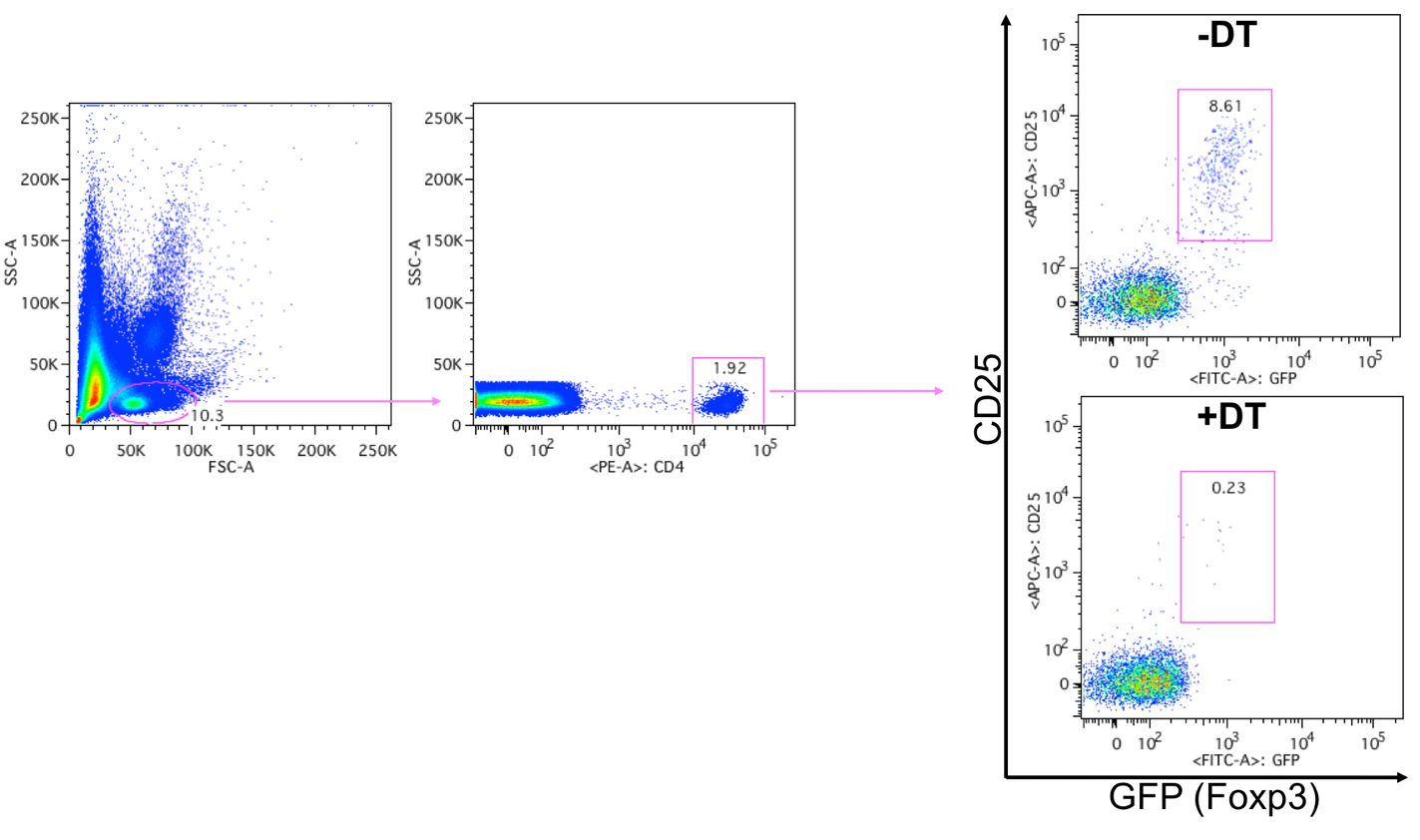
**

**Figure S2. Depletion of Foxp3^+^ T cells with DT:** Flow cytometry plots demonstrating the gating strategy used for the assessment of Foxp3^+^ T cell depletion by DT within the CD45RB^hi^ transfer model. Cells were first identified by their scatter and then gated based upon CD4 positivity. Cells were then gated on CD25 and GFP (Foxp3). The representative flow cytometry plots show the CD25^+^GFP^+^ population before DT (-DT) administration and after (+DT).

**Table S1** Real-time PCR Primer Sequences

| Target | Forward Sequences (5’–3’) | Reverse Sequences (5’–3’) |
| --- | --- | --- |
| TGF-β1 | CCCGAAGCGGACTACTATGC | CGAATGTCTGACGTATTGAAGAACA |
| TGF-β2 | CACCCAGCGCTACATCGATAG | CAGCGTCTGTCACGTCGAA |
| IL-10 | TTTGAATTCCCTGGGTGAGAA | GCTCCACTGCCTTGCTCTTATT |
| Foxp3 | ACTGGGGTCTTCTCCCTCAA | CGTGGGAAGGTGCAGAGTAG |
| Gapdh | GGTGGTCTCCTCTGACTTCAACA | GTTGCTGTAGCCAAATTCGTTGT |
